# Discrepancy between Mexican paediatricians and ESPGHAN experts regarding cow's milk allergy

**DOI:** 10.1002/jpr3.12147

**Published:** 2024-11-20

**Authors:** Fabian Hendricx, Emma Robert, Thomas Gestels, Hanne Delcourt, Benjamin Suarez Negroe, Rodrigo Vázquez‐Frias, Erick Manuel Toro Monjaraz, Josefina Monserrat Cazares Méndez, Carmen Ribes‐Koninckx, K. Huysentruyt, Yvan Vandenplas

**Affiliations:** ^1^ Vrije Universiteit Brussel (VUB), UZ Brussel, KidZ Health Castle Brussels Belgium; ^2^ Health Management Advisor Mexico City Mexico; ^3^ Hospital Infantil de México Federico Gómez (HIMFG), National Health Institute Mexico City Mexico; ^4^ Gastroenterology and Nutrition Department, Unit of Physiology and Gastrointestinal Motility National Institute of Pediatrics Mexico City Mexico; ^5^ Hospital Infantil Privado Star Médica and Hospital Angeles del Pedregal Mexico City Mexico; ^6^ Pediatric Gastroenterology and Paediatric Cystic Fibrosis Unit La Fe Hospital & La Fe Research Institute Valencia Spain

**Keywords:** CoMiSS, cow's milk‐related symptom score, infant, prevention, treatment

## Abstract

**Objectives:**

Cow's milk allergy (CMA) is a common food allergy in infants. Guidelines and recommendations for the diagnosis and management of CMA are based on scientific review of the available evidence. However, real‐world situations may oblige clinicians to adapt a different attitude.

**Methods:**

This paper evaluated the opinion of 42 Mexican paediatricians on the 73 statements presented in the recent position paper of the European Society of Paediatric Gastroenterology, Hepatology and Nutrition (ESPGHAN). Voting on the statements and their interpretation were identical for both groups (online and anonymous). Both groups were unaware of the other's outcome at the moment of the voting.

**Results:**

While the ESPGHAN group accepted all 73 statements, the Mexican group rejected 19 statements. Two rejections were due to the mean and median being below the predefined and agreed‐upon cut‐off, and 17 were due to over 75% of participants disagreeing with the statements. The greatest discrepancy was observed regarding the role of vitamin D in preventing CMA.

**Conclusion:**

While opinions on the prevalence, diagnosis and management of CMA were comparable between European paediatric gastroenterologists and Mexican general paediatricians for the majority of statements, significant differences were observed. It is essential to gather information from various regions and healthcare levels to enhance the impact of recommendations.

## Abstract (highlights)

**What is Known:**

- Cow's milk allergy (CMA) is the most prevalent food allergy during infancy.
- The diagnosis of CMA is debated because of the absence of specific symptoms and diagnostic tests, except for the oral food challenge which is often refused.
- As a consequence, there is a gap between the available evidence and the real‐world situation.

**What is New:**

- Non‐experts diagnose and manage CMA in a different way than experts.
- This study highlights and confirms significant differences in the diagnosis and management of CMA if ‘evidence‐based’ recommendations are compared to real‐world situations.
- The impact of information provided by companies on the knowledge and perception of non‐expert general paediatricians needs to be further investigated.

**Abbreviations**
AAF: amino‐acid formula
ATP: atopy patch test
BAT: basophil activation test
CI: confidence interval
CM: cow's milk
CMA: cow's milk allergy
CoMiSS: Cow's Milk‐related Symptom Score
EGID: eosinophilic gastrointestinal disorder
eHF: extensively (cow's milk‐based) hydrolysed formula
ESPGHAN: European Society of Paediatric Gastroenterology, Hepatology and Nutrition
FGID: functional gastrointestinal disorder
FPIAP: food protein‐induced allergic proctocolitis
FPIES: food protein‐induced enterocolitis syndrome
FSMP: foods for special medical purpose
GI: gastrointestinal
HRF: hydrolysed rice formula
IgE: immunoglobulin E
LCPUFA: long‐chain poly‐unsaturated fatty acid
OFC: oral food challenge
pHF: partially hydrolysed formula

## 1 INTRODUCTION

Cow's milk allergy (CMA) is one of the most prevalent food allergies during infancy, with a reported prevalence of oral food challenge (OFC) proven CMA of <1%.^1^ When less strict criteria are used, the prevalence is estimated between <1% and 4.9%. CMA can be immunoglobulin E (IgE)‐mediated, non‐IgE‐mediated or mixed. Depending on the type of immunological responses, the clinical manifestations are classified as immediate or delayed.^1^ Symptoms are not specific and an accurate diagnostic test outside the OFC is lacking.^1^ However, a correct diagnosis followed by appropriate management is crucial to prevent over‐ and under‐diagnoses and consequently over‐ and under‐treatment. Both over‐ and under‐diagnosis cause adverse effects on health and quality of life. A delay in CMA diagnosis can lead to faltering growth and malnutrition.^1^, ^2^

An expert group of the European Society of Pediatric Gastroenterology, Hepatology and Nutrition (ESPGHAN) has recently published a position paper outlining recommendations on the diagnosis and management of CMA.^1^ Since CMA and its management are significantly influenced by social contexts, eating habits and available resources, the purpose of this article is to assess the opinion of paediatricians from Mexico on the statements of the ESPGHAN paper to investigate regional differences.^1^

## 2 METHODS

The ESPGHAN expert group (*n* = 13) published a position paper on the diagnosis and management of CMA.^1^ The most important conclusions and recommendations were summarised in 73 statements (Table 1). These were circulated three times before the voting round was started. There was only one voting round, which was on‐line and anonymous. Each statement was given a score between 0 and 9. A score of 6 or more indicated agreement, while a score of 5 and less indicated disagreement. The higher the score, the greater the degree of agreement. Whenever the consensus was <75% (≥4 members voting <6), the statement was rejected.

**Table 1:** The list of statements that were voted on.

		ESPGHAN		Mexico	
		Median/median	Min–max no. of disagree	Mean/median	Min–max no. of disagree
1	Over‐diagnosis of CMA is common. The prevalence of authenticated CMA in infants and children is <1%.	9/9	9–9	**7/9**	**1–9**
			0		**12**
2	Within the GI tract, non‐IgE CMA can manifest with entities such as FPIAP, FPIES and eosinophilic GI disorders.	8.7/9	7–9	8.3/9	1–9
			0		4
3	FPIAP occurs mostly in breastfed infants, and is in most cases a benign, easily recognised condition that may not need treatment in some breastfed infants, depending on the severity and frequency of blood in the stools.	8.4/9	2–9	**6.4/8**	**1–9**
			1		**20**
4	Acute FPIES is a potential medical emergency whose accurate diagnosis remains a challenge and is based on symptoms and their timing.	8.8/9	8–9	8.2/9	1–9
			0		3
5	The diagnosis of FPIES is based on a clinical history of typical characteristic signs and improvement of symptoms after withdrawal of the suspected trigger food.	8.8/9	8–9	8.3/9	1‐9
			0		5
6	In case the history is unclear, but FPIES is suspected, other potential causes not related to CMA should be excluded and, if there is a favourable risk/benefit ratio, an OFC can be considered to help confirm the diagnosis.	8.8/9	7–9	8.2/9	1–9
			0		4
7	CMA is considered a possible factor in the pathogenesis of EGIDs.	8.9/9	7–9	8.3/9	5–9
			0		5
8	CMA is considered a possible factor in the pathogenesis of EoE, and where the index of suspicion is high, oesophageal biopsies should be taken while on a CM‐containing diet.	8.3/9	6–9	7.6/9	1–9
			0		9
9	Some patients with suspected FGIDs can improve with CM elimination regardless of CMA, but there are no specific tests to allow clarification of the diagnosis by discriminating between CMA and FGIDs.	7.8/9	4–9	**6.5/7**	**1–9**
			2		**18**
10	In patients not responding to conventional therapies for FGIDs, CMA can be considered, and patients trialled on a time‐limited elimination diet for 2–4 weeks, which should be followed by an OFC.	8.4/9	6–9	8.4/9	1–9
			0		3
11	In patients not responding to conventional therapies for (GOR(D)), CMA can be considered, and patients trialled on a time‐limited elimination diet for 2–4 weeks, which should be followed by an OFC.	8.8/9	8–9	8.4/9	1–9
			0		4
12	In infants who present with crying and irritability, there is insufficient data to recommend a time‐limited CM elimination diet for 2–4 weeks followed by an OFC.	8.4/9	6–9	7.0/8	1–9
			0		15
13	There is insufficient data to support infant colic occurring as a single manifestation of CMA.	8.4/9	6–9	7.7/9	1–9
			0		6
14	When treatment for infant colic, fulfilling Rome IV clinical research criteria, is considered, and where CMA is suspected based on additional symptoms, a time‐limited elimination diet for 2–4 weeks can be trialled, which should be followed by an OFC.	7.9/9	4–9	8.4/9	1–9
			1		2
15	In patients not responding to conventional therapies for constipation, including laxatives in optimal dosage, CMA can be considered, and a time‐limited elimination diet for 2–4 weeks can be started, which should be followed by an OFC.	7.9/8	6–9	7.6/9	1–9
			0		8
16	There is insufficient evidence regarding a higher risk of infectious disease in infants with CMA.	8.3/9	5–9	**6.7/8.5**	**1–9**
			1		**15**
17	Absence of family history does not exclude the possibility of CMA.	8.8/9	8–9	8.7/9	5–9
			0		2
18	Environmental factors (e.g., pollution, antibiotic (over‐)use) are possible risk factors for CMA.	7.8/8	4–9	7.6/9	1–9
			3		7
19	The baseline CoMiSS and its reduction during an elimination diet may be indicative for CMA, but is not diagnostic.	8.4/9	6–9	7.7/9	1–9
			0		7
20	The response to a diagnostic elimination diet followed by an OFC is the corner stone for the diagnosis of CMA.	8.9/9	8–9	8.0/9	1–9
			0		7
21	In rare cases when CMA is suspected in an exclusively breastfed infant, a diagnostic maternal CM‐free diet for 2–4 weeks while continuing to breastfeed may be considered. To confirm the diagnosis, CM should then be reintroduced in the maternal diet with monitoring of symptoms.	8.8/9	8–9	7.5/9	1–9
			0		8
22	In formula‐fed infants, a CM‐derived eHF is the first choice for a diagnostic elimination diet.	7.2/9	0–9	7.7/9	1–9
			2		8
23	Only CM‐derived eHFs tested in randomised clinical trials should be used.	8.6/9	7–9	**7.2/9**	**1–9**
			0		**12**
24	There are insufficient comparative trials to make a recommendation whether to use whey versus casein hydrolysates.	8.8/9	8–9	**6.2/7**	**1–9**
			0		**18**
25	In patients with CMA and severe diarrhoea and/or with severe malnutrition, the transient use of a formula without lactose for 2–4 weeks may be preferred.	7.0/8	0–9	**5.0/5**	**1–9**
			3		**24**
26	In formula‐fed infants, AAF for a diagnostic elimination diet should be reserved for severe cases or patients with severe malnutrition.	8.5/9	7–9	7.3/9	1–9
			0		10
27	Although some consensus papers recommend a step‐down approach using AAF as diagnostic elimination diet in every infant suspected of CMA, there is insufficient evidence for this recommendation.	8.6/9	6–9	**6.1/7**	**1–9**
			0		**17**
28	Although less studied than CM‐based eHFs, HRFs can be considered as an alternative for a diagnostic elimination diet.	7.4/8	1–9	8.3/9	5–9
			2		5
29	Soy infant formula should not be used as the first choice for the diagnostic elimination diet but can be considered in some cases for economic, cultural and palatability reasons.	7.6/9	0–9	7.8/9	1–9
			1		6
30	In IgE‐mediated allergy, the response to the diagnostic elimination diet is to be expected within 1–2 weeks.	8.8/9	8–9	7.9/9	1–9
			0		7
31	In non‐IgE mediated allergy, the response to the diagnostic elimination diet is to be expected within 2–4 weeks.	8.7/9	7–9	7.4/9	1–9
			0		10
32	A DBPCFC is the gold standard for confirming a diagnosis of CMA.	8.8/9	8–9	7.7/9	5–9
			0		6
33	In clinical practice, the open OFC is clinically more feasible and practical than DBPCFC and is sufficient to confirm the diagnosis of CMA and the development of oral tolerance.	8.7/9	7–9	7.5/9	1–9
			0		10
34	In IgE‐mediated CMA, the OFC test should be supervised by trained medical healthcare professionals.	8.8/9	7–9	8.7/9	5–9
			0		1
35	The DBPCFC is recommended for unclear cases and research purposes.	8.8/9	8–9	7.7/9	1–9
			0		10
36	The result of a negative DBPCFC should be followed by an OFC of a regular age‐appropriate serving to exclude delayed reactions.	8.4/9	7–9	7.5/9	**5–9**
			0		**13**
37	If an elimination diet is not effective in reducing symptoms and/or the OFC is unable to reproduce symptoms, the diagnosis of CMA cannot be made.	8.8/9	7–9	**6.3/8**	**1–9**
			0		**16**
38	Elevation of total IgE does not generally contribute to the diagnosis of CMA.	8.8/9	8–9	7.4/9	1–9
			0		9
39	Elevated sIgE and SPT show sensitisation to CM protein, but do not confirm CMA, whose diagnosis is based on the presence of symptoms.	8.8/9	8–9	7.5/9	1–9
			0		9
40	The NPVs of sIgE and SPT are high when evaluating IgE‐mediated allergy.	8.5/9	7–9	7.4/8.5	1–9
			0		10
41	The APT is not recommended for the routine diagnosis of non‐IgE mediated CMA mainly due to insufficient evidence for reproducibility and efficacy.	8.6/9	6–9	8.5/9	5–9
			0		2
42	Currently, component‐resolved diagnostics and BAT are not recommended for the routine diagnosis of CMA due to insufficient evidence for reproducibility and efficacy.	8.8/9	8–9	7.8/9	1–9
			0		9
43	There is insufficient evidence to recommend routine upper or lower GI endoscopy for diagnosing CMA because of lack of specificity of histological findings.	9/9	9	8.1/9	2–9
			0		6
44	IgG‐antibodies against CM protein and biomarkers such as calprotectin, alpha‐1‐antitrypsin, beta‐defensin and tests such as the allergen‐specific lymphocyte stimulation test, and determination of thymus and activation‐regulated chemokines are not indicated in the routine diagnosis of CMA.	8.8/9	8–9	7.5/9	1–9
			0		10
45	Professional dietary counselling should be offered to mothers on CM elimination diets. Supplements of calcium and vitamin D are recommended for lactating mothers.	8.8/9	8–9	8.6/9	5–9
			0		3
46	Complementary feeding should be introduced at the same age as in children without CMA. The introduction of foods should follow the same recommendations as for those without CMA, except for dairy.	8.8/9	7–9	8.4/9	5–9
			0		3
47	Dietary monitoring of an adequate intake of macro‐ and micronutrients, particularly vitamin D and calcium, is required in children on a CM elimination diet especially in those older than 1 year of age.	9/9	9	8.7/9	5–9
			0		2
48	As CM exclusion diets could be associated with micronutrients and growth deficiencies, close dietary monitoring is essential, especially after the introduction of complementary feeding.	8.8/9	8–9	8.8/9	5–9
			0		2
49	Professional dietary counselling by a dietitian should be offered to children on CM elimination diets to prevent malnutrition and promote a varied diet leading to normal feeding behaviour.	8.8/9	7–9	8.4/9	1–9
			0		3
50	Close monitoring of growth is mandatory in children with CMA as they may suffer from growth faltering.	8.8/9	8–9	8.7/9	5–9
			0		2
51	We recommend to use only FSMPs, such as eHFs and AAFs, for which appropriate growth and nutritional studies have been published.	8.8/9	7–9	**7.2/9**	**1–9**
			0		**12**
52	In formula‐fed infants, a CM‐derived eHF is the first choice for a therapeutic elimination diet.	7.8/9	0–9	**7.2/9**	**1–9**
			1		**12**
53	There is insufficient evidence demonstrating that the addition of pro‐, pre‐ or synbiotics studied so far to eHFs improves their therapeutic efficacy.	8.9/9	8–9	**6.5/8**	**1–9**
			0		**14**
54	pHFs are not recommended in the treatment of CMA.	8.8/9	7–9	**7.2/9**	**1–9**
			0		**12**
55	Regarding the therapeutic elimination diet, AAF should be reserved for severe cases or infants with an absent or partial response to eHF.	8.3/9	1–9	7.8/9	1–9
			1		7
56	HRFs can be considered as an alternative to CM‐derived eHF for therapeutic elimination diet.	7.8/9	5–9	8.3/9	5–9
			2		5
57	If a diagnostic elimination diet followed by OFC has shown efficacy of a soy infant formula, such a formula can be considered as an alternative for a therapeutic elimination diet for economic, cultural and/or palatability reasons.	7.6/8	0–9	7.8/9	1–9
			1		6
58	The OFC after the first period of therapeutic elimination diet can be done in a similar fashion to that after the diagnostic elimination diet or according to the milk ladder, starting with small amounts of baked milk (e.g., milk‐containing biscuits).	8.8/9	8–9	7.3/9	1–9
			0		11
59	Standardisation of the home challenge by applying the milk ladder adapted to local dietary habits is recommended.	8.8/9	8–9	7.9/9	1–9
			0		7
60	The provision of oral immune therapy in selected patients with persistent IgE‐mediated CMA should be limited to specialised centres.	8.8/9	8–9	7.9/9	1–9
			0		5
61	Breastfeeding should be promoted for its multiple benefits, although its preventive effect on CMA has not been consistently documented.	9/9	9	7.2/9	1–9
			0		11
62	Dietary restrictions, other than those warranted for the pregnant woman herself, are not indicated during pregnancy and lactation to prevent CMA.	9/9	9	7.9/9	1–9
			0		6
63	There is no convincing scientific evidence that the avoidance or delayed introduction of CM‐based formula reduces or increases the risk of CMA in infants considered at high risk of allergic diseases.	8.4/9	4–9	7.3/9	1–9
			1		11
64	It remains unclear whether avoiding regular consumption of CM‐based formula during early life reduces the risk of CMA in children.	8.5/9	6–9	8.1/9	1–9
			0		6
65	Feeding supplementation (i.e., providing any kind of formula beyond breast milk) during the first days of life is not recommended for the prevention of CMA.	8.8/9	5–9	8.2/9	3–9
			1		7
66	For infants with a documented family history of allergic disease who cannot be exclusively breastfed, there is insufficient evidence to recommend the routine use of pHF, eHF‐Whey and eHF‐Casein for preventing CMA.	8.3/9	4–9	6.6/9	**1–9**
			1		**15**
67	The role of HRF for preventing CMA has not been studied.	8.8/9	7–9	8.4/9	4–9
			0		5
68	For infants with a documented family history of allergic disease who cannot be exclusively breastfed, there is no evidence to recommend soy formula for preventing CMA.	8.5/9	7–9	**6.7/9**	**1–9**
			0		**13**
69	There is insufficient evidence to recommend the use of pro‐, pre‐ or synbiotics studied so far for CMA prevention.	8.8‐9	7–9	**6.8/8.5**	**1–9**
			0		**15**
70	There is insufficient evidence to recommend the use of LCPUFAs for CMA prevention.	8.8/9	7–9	**6.8/9**	**1–9**
			0		**14**
71	Vitamin D supplementation has no role in CMA prevention.	8.8/9	7–9	**5.4/5**	**1–9**
			0		**22**
72	The choice of formula for the treatment of CMA should take into consideration cost and availability of the therapeutic formula.	8.8/9	8–9	7.9/9	1–9
			0		9
73	CMA may lead to substantial impairments in quality of life, both for the children and their caregivers.	8.8/9	8–9	8.4/9	2–9
			0		3

Abbreviations: AAF, amino acid‐based formula; APT, atopy patch test; BAT, basophil activation test; CM, cow's milk; CMA, cow's milk allergy; CoMiSS, Cow's Milk‐related Symptom Score; DBPCFC, double‐blind placebo‐controlled food challenge; EGID, eosinophilic gastrointestinal disorder; eHF, extensively hydrolysed formula; EoE, eosinophilic oesophagitis; ESPGHAN, European Society of Paediatric gastroenterology, Hepatology and Nutrition; FGID, functional GI disorder; FPIAP, food protein‐induced allergic proctocolitis; FPIES, food protein; FSMP, Foods for Special Medical Purpose; GI, gastrointestinal; GOR(D), gastro‐oesophageal reflux (disease); HRF, hydrolysed rice formula; IgE, immunoglobulin E; IgG, immunoglobulin G; LCPUFA, long‐chain polyunsaturated fatty acid; NPV, negative predictive value; OFC, oral food challenge; pHF, partially hydrolysed formula; sIgE, specific IgE; SPT, skin prick test.

The same statements were submitted to 42 Mexican colleagues (mainly general paediatricians [*n* = 30], paediatric gastroenterologists [*n* = 8] and neonatologists [*n* = 4]). They were all invited to participate in a 2‐day course on infant nutrition organised by a formula company, but they were contacted about the voting for the statements before the meeting. Voting was anonymously, on‐line. The Mexican colleagues were not aware of the results of the voting by the ESPGHAN members, because the ESPGHAN paper was not yet published.

The interpretation of the voting was identical for the European and Mexican groups: ≥6 means agreement; disagreement if score ≤5 or if ≥11 members voted <6 (or <75% who voted >6).

## 3 RESULTS

While the ESPGHAN group accepted all 73 statements, 10 statements were rejected by the Mexican group (2 of the 10 were rejected because the mean and median were <6 and 8 of the 10 rejections were because >75% of the participants voted <6. The greatest discrepancy was observed regarding the role of vitamin D in the prevention of CMA.

A more detailed interpretation of the voting is listed below:

### 3.1 Statement 1

The prevalence of CMA is still debated. According to the EuroPrevall data, the incidence of CMA was estimated at 0.54%, but with large variation in the incidence of non‐IgE‐mediated allergy across countries. In the United Kingdom, more children presented with non‐IgE‐mediated CMA (56.3%) than IgE‐mediated CMA (43.7%), while the prevalence of non‐IgE‐mediated CMA was not reported in many countries.^3^ According to a recent publication, the overall pooled self‐reported lifetime prevalence of CMA was 5.7% (95% confidence interval [CI]: 4.4–6.9), while the point prevalence of food‐challenge‐verified allergy for cow's milk (CM) was 0.3% (0.1–0.5).^4^ While limiting prevalence data to challenge‐proven CMA will underestimate the prevalence since many parents refuse a challenge test, a self‐reported prevalence will overestimate the latter.

### 3.2 Statement 2

There was consensus that non‐IgE CMA can manifest with entities such as food protein‐induced allergic proctocolitis (FPIAP), food protein‐induced enterocolitis syndrome (FPIES) and eosinophilic gastrointestinal disorders (EGIDs).

### 3.3 Statement 3

Twenty Mexican doctors consider that breastfed infants presenting with FPIAP should be put on a CM‐free elimination diet by continuing breastfeeding with a mother on a CM‐free diet. Mucous or green‐coloured stools should not be considered as symptoms suggesting CMA in otherwise healthy infants. The prevalence of FPIAP ranges from 0.16% in healthy children up to 64% in patients with haematochezia.^5^, ^6^, ^7^ In most cases, FPIAP sets off within the first weeks of life and resolves in late infancy. Whether treatment of FPIAP is needed or not is debated.^8^, ^9^ In exclusively breastfed infants, FPIAP in the absence of other atopic symptoms should be managed by observation of the symptoms without dietary intervention during the first month of haematochezia as it is generally a benign and a self‐limiting disorder despite marked mucosal abnormality on endoscopy.^9^ CMA diagnosis should only seldom be considered in exclusively breastfed infants with chronic symptoms.^10^ Whether a diagnostic elimination diet should be started in formula‐fed infants is debated since a large cohort study reported that CM FPIAP was associated with an increased risk of developing IgE‐CMA (with adjusted odds ratio 5.4 (95% CI: 1.4–20.8)) and raised concerns about the potential role of delayed introduction in IgE‐CMA development in this vulnerable population.^11^, ^12^ However, reintroduction of CM is always recommended.

### 3.4 Statements 4–6

A minority of the Mexican doctors disagreed with the statements on FPIES.

### 3.5 Statement 7

Also, a minority of Mexican doctors were in moderate disagreement that CMA could be the cause of EGIDs.

### 3.6 Statement 8

Nine of the 42 Mexican colleagues disagreed with the statement on eosinophilic esophagitis, reaching a full agreement with the European group.

### 3.7 Statements 9–11 and 15

The prevalence of CMA in infants with functional gastrointestinal disorders (FGIDs) is controversial. The opinion of the Mexican group regarding the complex relation between CMA and FGIDs or disorders of gut–brain interaction (gastro‐oesophageal reflux, constipation) differs slightly from the voting result of the ESPGHAN group. Although the mean and median did not differ, about 20% of the Mexican participants consider each FGID as possibly CMA‐related. Gastrointestinal (GI) symptoms may be driven by an interplay of factors such as esophagitis and GI inflammation, dysmotility, visceral hyperalgesia, and dysbiosis.^13^ In some infants, food allergens play a role as triggers for FGIDs that occur in association with other GI, respiratory, or skin manifestations as well as poor growth.^14^, ^15^ The exclusion of CM from the diet of infants with common GI symptoms such as regurgitation, constipation and infant colic, without an established diagnosis of CMA, induces the risk of establishing a false diagnosis of CMA.^6^

### 3.8 Statements 12–14

While there is consensus that when the Rome IV clinical research criteria for colic are fulfilled, a time‐limited elimination diet and OFC may be considered, Mexican doctors are also in favour to propose a similar approach when infants present with crying and distress as single manifestation, even if the Rome IV diagnostic criteria are not fulfilled.

### 3.9 Statement 16

In contrast to the European group, 36% of the Mexicans considered that there is evidence that infants with CMA have a higher risk of acquiring an infectious disease.

### 3.10 Statements 17 and 18

There is consensus that CMA can develop in the absence of a family history of atopic disease. The appreciation of the possible role of environmental factors regarding the risk of developing allergic disorders was comparable.

### 3.11 Statement 19

While the ESPGHAN group accepted unanimously that Cow's Milk‐related Symptom Score (CoMiSS) is a (useful) awareness tool, this statement was rejected by 7 of the 42 Mexican participants. It is not clear if this is because they did not know about CoMiSS or because they consider it to be a diagnostic tool.

### 3.12 Statements 20 and 21

The prevalence of CM‐related symptoms in breastfed infants is debated nowadays, and beyond the scope of this manuscript. Seven of the 42 Mexicans disagreed with the statement that ‘the response to a diagnostic elimination diet followed by an OFC is the cornerstone for the diagnosis of CMA’, despite the fact that this definition is proposed in all guidelines and recommendations. The reason for the disagreement is the fact that many parents refuse the OFC as it will make the child sick again if positive. Consequently, a similar proportion disagreed with continuing breastfeeding, putting the mother on a CM‐free diet and reintroducing CM in the diet of the mother.

### 3.13 Statements 22–25

Fifteen per cent (2 of the 13) and 21% (9 of the 42) in the ESPGHAN and Mexican groups, respectively, disagreed with the statement that an extensively CM‐based hydrolysed formula (eHF) should be the first option in the management of CMA in formula‐fed infants. Although all European participants estimated that only tested eHFs can be recommended, 29% of the Mexicans disagreed with this statement. Even more striking, while there was broad consensus in Europe that no recommendation can be made for whey versus casein eHF, 43% (18 of the 42) of the Mexican participants disagreed with this statement. The statement that in case of severe diarrhoea, a 2–4 lactose‐free diet is recommended was rejected by the Mexican group, while this statement was accepted in Europe. These differences in opinion regarding the choice of diagnostic and therapeutic formula illustrate the impact of differences in the availability of products in different regions of the world. It also suggests the impact of information provided by the companies.

### 3.14 Statements 26 and 27

While all European members consider that amino‐acid‐based formula (AAF) should be reserved for severe cases or patients with severe malnutrition, 10 of the 42 (23%) Mexicans disagreed with this statement. A systematic step‐down approach starting with AAF as diagnostic elimination diet was unanimously rejected by ESPGHAN, but 17 of the 42 (40%) Mexicans were in favour of a step‐down approach.

### 3.15 Statement 28

A minority in Europe (2 of the 13, 15%) and Mexico (5 of the /42, 12%) rejected the recommendation to use a hydrolysed rice formula (HRF) as diagnostic elimination diet.

### 3.16 Statement 29

The voting about the use of soy infant formula as diagnostic elimination diet was comparable between both groups.

### 3.17 Statements 30 and 31

Some Mexican participants disagreed with the proposed duration of the diagnostic elimination diet: 1–2 weeks in IgE and 2–4 weeks in non‐IgE mediated CMA.

### 3.18 Statements 32–37

The voting on the statements concerning the use of the OFC as the best diagnostic criterion illustrates the more practical approach of the Mexican participants, which may be related to the healthcare organisation as well. Sixteen of the 42 (38%) Mexicans disagreed with the statement that ‘if an elimination diet was not effective in reducing symptoms and/or the OFC unable to reproduce symptoms, the diagnosis of CMA cannot be made’.

### 3.19 Statements 38–44

Regarding testing for CMA (IgE and others), disagreement was less frequent, although overall Mexican participants tended to be more open for non‐classic tests such as IgG.

### 3.20 Statements 45–50

The Mexican and European voting regarding the recommendations to closely monitor the nutrition of infants with CMA, including the guidance by a dietician, is very comparable.

### 3.21 Statement 51

Twelve of the 41 (29%) Mexicans disagree with the statement that only foods for special medical purposes (FSMPs), such as ETFs and AAFs, for which appropriate growth and nutritional studies have been published, should be used.

### 3.22 Statement 52

Twelve of the 42 (29%) Mexicans disagree that a CM‐based eHF should be considered as the preferred option for a therapeutic elimination diet.

### 3.23 Statement 53

Fourteen of the 42 (33%) Mexican doctors consider there is sufficient evidence that the addition of pro‐, pre‐ or synbiotics to eHFs improves their therapeutic efficacy.

### 3.24 Statement 54

Twelve of the 42 (29%) Mexicans disagree with the statement that pHFs should not be used in the management of CMA. This discrepancy may be related to ‘misdiagnosis’ (considering an FGID or disorder of gut–brain interaction as CMA) or related to acceptability and cost. The disagreement for statements 51–54 illustrates again how regional differences in healthcare organisation and availability of products influence the answers to the statements. The influence of information provided by companies is also likely to contribute to the differences in opinion regarding the supplementation of pre‐, pre‐ and synbiotics to eHFs.^16^, ^17^

### 3.25 Statements 55–57

The opinion to reserve AAF to severe cases was comparable with 13% and 16% rejection, respectively. Mexican doctors tend to be more convinced about the efficacy of HRF than European voters. There was no difference regarding the use of soy as therapeutic formula (13% rejection in Europe vs. 14% in Mexico).

### 3.26 Statements 58 and 59

The quite significant disagreement among the Mexican colleagues compared to the Europeans regarding the diagnostic OFC, is a logical consequence that there is also a different opinion regarding the OFC after the first period of the therapeutic elimination diet. The ‘milk ladder’ was more accepted than the OFC.

### 3.27 Statement 60

There is consensus that oral immune therapy should be reserved for specialised centres.

### 3.28 Statements 61 and 62

In contrast to the unanimity in the ESPGHAN group that there is no evidence‐based preventive effect of exclusive breastfeeding on CMA, 26% of the Mexican colleagues consider there is. There is a better consensus regarding the recommendation that mothers should not have any dietary restrictions during breastfeeding.

### 3.29 Statements 63–65

Opinions regarding the effects of the timing of introduction of CM formula in breastfed infants differed between both groups.

### 3.30 Statements 66–71

While only 1 of the 13 (8%) from the ESPGHAN group disagreed with the statement that there is insufficient evidence to recommend hydrolysates for the prevention of CMA, 15 of the 42 (36%) Mexican colleagues did not accept this statement. This disagreement illustrates the ongoing debate regarding prevention. While the position papers of ESPGHAN and the guideline of the European Academy of Allergology and Clinical Immunology do not recommend in favour or against the use of pHF,^1^, ^18^ still many papers suggest efficacy.^19^, ^20^, ^21^ Regarding the efficacy of pre, pro‐ and synbiotics studied so far, and long chain poly unsaturated fatty acids in the prevention of CMA, opinions differed clearly. The largest discrepancy was observed regarding the statement that vitamin D has no role in CMA prevention: while this statement was unanimously accepted in the ESPGHAN group (1 vote ‘7’ and 12 times ‘9’), this statement was rejected in the Mexican group (mean and median <6).^16^, ^17^

### 3.31 Statement 72

Cost and availability of formula are factors to be taken into consideration, although 21% of the Mexicans disagreed.

### 3.32 Statement 73

Finally, there was consensus that CMA does have a significant impact on the quality of life.

## 4 DISCUSSION

Thirteen European paediatricians, all members of ESPGHAN, agreed on 73 statements regarding the diagnosis, prevention and management of CMA.^1^ Forty‐two Mexican paediatricians, mainly general paediatricians, voted on the same 73 statements, but disagreed on 19 (26%), based on the fact that <75% agreed with the statement (≥11/42 voted voting <6). For the two statements, the mean and median scores for the statement were <6.

Twelve of the 42 (29%) Mexican group consider that the prevalence of CMA is above 1%, and are thereby in agreement with a substantial part of the literature suggesting that the prevalence of CMA is between 1.9% and 4.9%.^22^ While ESPGHAN considered that some patients with suspected FGIDs can improve with CM elimination regardless of CMA in the absence of specific tests to allow clarification of the diagnosis by discriminating between CMA and FGIDs, the Mexican group strongly disagreed with this statement. While ESPGHAN considered that there is insufficient evidence regarding a higher risk of infectious disease in infants with CMA, the Mexican group voted differently. There is indeed some literature suggesting that infants with CMA are at higher risk for infectious diseases. However, this literature suggests associations and not necessarily causality. It is likely that the difference in voting can be explained by a different interpretation of association and causality.

In contradiction to the European point of view, the Mexican doctors consider that the hydrolysates do not need clinical testing in a randomised controlled trial before being used in the clinic. The Mexican group disagreed with the statement that there is insufficient data to recommend whey or casein hydrolysates. Given the fact that Mexico is mainly using products from the United States, it can be hypothesised that casein hydrolysates are preferred. The Mexican colleagues also rejected (17 of the 42; 40%) the statement to not endorse a step‐down approach using AAF as diagnostic elimination diet in every infant suspected of CMA.

Although both groups strongly endorsed that the OFC is the golden standard for the diagnosis of CMA, the Mexican group rejected two statements: (i) a negative double‐blind placebo‐controlled food challenge (DBPCFC) should be followed by an OFC of a regular age‐appropriate serving to exclude delayed reactions, and (ii) if an elimination diet was not effective in reducing symptoms and/or the OFC is unable to reproduce symptoms, the diagnosis of CMA cannot be made.

As a logical consequence of the previous voting, the Mexican group also rejected the statement that only hydrolysates and amino acid formulas with documented data on growth and nutrition should be used, probably considering that all products in the market contain all components required. Since the group was in favour of a step‐down approach, they also rejected that an eHF should be the first therapeutic option. Also, the Mexican group considers there is sufficient data to conclude that supplementation of therapeutic formula with ‘biotics’ improves its therapeutic efficacy. Somehow in contradiction with the step‐down approach recommendation, the group also considers that pHF may be used in CMA. We hypothesise that this voting is based on the studies showing that 50% of the infants with proven CMA do tolerate a pHF.^23^

The Mexican group rejected all statements regarding prevention. According to the Mexican group, there is evidence that hydrolysates (pHF, eHF) can be recommended to reduce the allergy risk. The group considers also that soy formula can be used, and that ‘biotics’, long‐chain polyunsaturated fatty acids (LCPUFAs) and vitamin D reduce the risk to develop allergic disease.

One of the limitations of this study is that being a survey, there may be bias in the responses. However, it is important to consider the perspectives of both approaches in treating and resolving a condition as common as CMA. The impact of information provided by companies on the knowledge and perception of paediatricians needs to be further investigated. This impact may explain some of the differences observed in the responses.

## 5 CONCLUSIONS

We conclude that while the opinion regarding the prevalence, diagnosis, management and prevention of CMA between European paediatric gastroenterologists and Mexican general paediatricians was comparable in the majority of the statements, important differences were observed which have an important impact, especially on CMA diagnosis and management practices. It should be considered to collect information from different parts of the world and different levels of healthcare to evaluate the impact of recommendations.^1^ We also conclude that is very important to keep updated on the latest diagnosis and treatment tools to benefit the clinical practice.

## CONFLICTS OF INTEREST STATEMENT

Yvan Vandenplas has participated as a clinical investigator, advisory board member, consultant, and/or speaker for Abbott Nutrition, Alba Health, Arla, Ausnutria, Biogaia, Danone, ELSE Nutrition, Friesland Campina, Nestle Health Science, Nestle Nutrition Institute, Nutricia, Pileje, Sanulac and United Pharmaceuticals (Novalac). Rodrigo Vázquez‐Frias has participated as a clinical investigator, advisory board member, consultant, and/or speaker for Abbott Nutrition, Nestle Nutrition Institute, Mead Johnson Nutrition, Sanulac and Stendhal. The remaining authors declare no conflicts of interest.
